# Efficacy of Paclitaxel Combined with Kanglaite Injection in Treatment of Bone Metastases of Lung Cancer

**Published:** 2019-08

**Authors:** Liming CAO, Long LONG, Chengping HU

**Affiliations:** 1. Department of Respiration, Xiangya Hospital, Central South University, Changsha, 410008, P.R. China; 2. Department of Cardio-Thoracic Surgery, Xiangya Hospital, Central South University, Changsha, 410008, P.R. China

**Keywords:** Paclitaxel, Kanglaite, Lung cancer bone metastasis, Mouse, Efficacy

## Abstract

**Background:**

We aimed to investigate the efficacy of paclitaxel combined with kanglaite injection in the treatment of bone metastases of lung cancer in mice.

**Methods:**

Human lung cancer cell line A549 was inoculated into 100 C57BL/6 mice to establish bone metastasis model of lung cancer. Eighty successful modeling mice were randomly divided into four groups: Kanglaite (group A), Paclitaxel (group B), Paclitaxel combined with kanglaite (group C) and 0.9% sodium chloride solution (control group), 20 in each group. The mice started taking drugs on the 5th day after inoculation. The treatment lasted for 21 days, and the changes of body weight were observed. Evaluation of the efficacy of drug therapy was performed by comparing the pain behavior of the treated mice with that of the control mice.

**Results:**

The physical improvement rates in group A and group B were lower than that in group C (*P*<0.05). The bone metastasis area and tumor weight in group A, group B and group C were significantly lower than those in control group after 21 days of treatment (*P*<0.05). The tumor area and tumor weight in group C were significantly lower than those in group A and group B (*P*<0.05).

**Conclusion:**

Paclitaxel combined with kanglaite is more effective than paclitaxel or kanglaite alone in improving bone metastasis of lung cancer and has an important significance in clinical treatment of bone metastasis of lung cancer.

## Introduction

Bone metastasis of lung cancer is one of the diffusive conditions of cancer cells in the advanced stage of lung cancer (1). The incidence of lung cancer metastasis is very high, and there are 50%∼70% probability of bone metastasis which is one of the leading causes of lung cancer death and can seriously affect the quality of life of patients during its onset (2, 3).

Chemotherapy is commonly used for bone metastasis of lung cancer, that is, cancer drugs were used to relieve pain in patients (4). Paclitaxel and kanglaite are common anticancer drugs in cancer chemotherapy. Paclitaxel has reliable anticancer activity (5). Kanglaite, an effective component extracted from semen coicis, can inhibit cancer cells and improve the immune function of patients (6). Paclitaxel and kanglaite are commonly used in chemotherapy for bone metastasis of lung cancer (7–9).

The mechanism of bone metastasis of lung cancer is complicated, and the general anti-tumor chemotherapeutic drugs have some side effects, such as toxic reaction, bone marrow suppression and so on. Therefore, anti-tumor chemotherapeutic drugs should be carefully selected in clinical application (10–12).

Human lung adenocarcinoma cell line A549 (13) was inoculated into healthy C57BL/6 mice for the establishment of bone metastasis model of lung cancer in this study to investigate the efficacy of paclitaxel combined with kanglaite in the treatment of bone metastasis of lung cancer in mice, and to provide information for its application in bone metastasis of lung cancer.

## Materials and Methods

### Main materials of the experiment

One hundred males C57BL/6 mice in SPF grade, with an age of (5∼9) weeks and an average weight range of (210.94 ±10.45) g (Institute of Experimental Animals, Chinese Academy of Medical Sciences, Beijing) were raised in SPF grade animal house of Longhua Hospital Affiliated to Shanghai University of Traditional Chinese Medicine, China from 2017 to 2018 at (23±2) °C room temperature with free food and free drinking water. Human lung cancer cell line A549 was purchased from Animal Center of Shanghai Pharmaceutical Engineering Institute.

The study was approved by the Ethics Committee of Xiangya Hospital, Central South University.

Thirty mg/5 ml paclitaxel (Yangtze River Pharmaceutical Group Co., Ltd. SFDA Approval No. H20058719); 10 g/100 ml kanglaite injection (Zhejiang Kanglaite Pharmaceutical Co., Ltd. SFDA Approval No. Z10970091); Penicillin (Baxter Company). Instruments: USA PXi XRAD 320 small animal irradiator (Shanghai Heyi instrument Co., Ltd); Small animal gas anesthetic machine SA428 (Jiangsu Sans biotech Co., Ltd.).

### Establishment of mouse model and treatment

Establishment of lung cancer bone metastasis mouse model (14). After resuscitation of human lung cancer cell line A549 frozen in liquid nitrogen, A549 cells in exponential growth phase were collected. After digested by trypsin, the cells were prepared into 1×10^7^/ml A549 cell dilution with normal saline, and only the upper suspension was used. The mice were anesthetized by small animal anesthetic machine, then dressed and sterilized. Overall, 150 μl cell suspension was obtained by injection needle and injected into tibial plateau of mice. Each mouse was injected penicillin 1×10^5^ U intraperitoneally every day 3 days after operation. After operation, the mice were kept in laminar flow cabinet to observe their living states.

### Treatment

One hundred SD mice were scanned by radioisotope scanning and the radionuclide aggregation site was as bone metastasis positive. Eighty mice models of bone metastasis of lung cancer were established successfully and randomly divided into group A, group B, group C and control group according to different drug regimen. Group A was treated with paclitaxel injection; Group B was treated with kanglaite injection; Group C was treated with paclitaxel combined with kanglaite injection. The control group was injected with sodium chloride injection. There were 20 rats in each group and the treatment lasted for 21 days. Mice in group A were intragastrically perfused with 10 mg/kg paclitaxel (Paclitaxel injection diluted with 25 ml saline to 1 mg/ml concentration). Mice in group B were intraperitoneally injected with 0.006 g/10 g kanglaite once every 3 days for 5 times. Mice in group C were intragastrically perfused with 10 mg/kg paclitaxel and injected kanglaite at the same time (same as group A). Mice in the control group were only given 0.9% sodium chloride injection. (Note: The dosages were calculated according to the reduction method of human and mouse body surface area).

### Observation index

The changes of body weight before and after modeling were observed. Evaluation of the efficacy of drug therapy was performed by comparing the pain behavior of the treated mice with that of the control mice, improvement of the pain behavior was considered as effective treatment. Taking the average value of pain measurement in the control group as the standard, the physical improvement of the mice after treatment was compared (Area above threshold of struggle: 7348.6 mv·s; Accumulated immobile time: 103.6 s; Single accumulated immobile time: 60.5 s). The tumor growth was observed in real time by Fluor Vivo imaging system, and the area of bone metastases of lung cancer in mice and tumor weight of mice after 21 days treatment was compared.

### Observation of pain behavior

Detection of paw withdrawal latency (PWL) (15): In the observation cage, the lights were gathered in the middle of the toe floor of the mice after 21 days of treatment, and the upper limit value of PWL was set at 20 s to avoid scalding. The latent period from the beginning of irradiation to the leg lift or escape of the mice was taken as PWL, and the average value was taken for 3 times, at intervals of 10 min.

Determination of paw withdrawal threshold (PWT) (16). After 21 days of treatment, the rat paws were stimulated by von Frey filaments in different thresholds, and the highest threshold was 26 g. A rapid paw withdrawal during stimulation or while removing the filaments was considered paw withdrawal positive. Three times positive reaction was tested in 5 consecutive times by one von Frey filament, then it was taken as PWT threshold, the interval of each experiment was 10 s. The times of spontaneous paw withdrawal were observed. (The mice moved freely in the glass box. Walking gait and times of spontaneous paw withdrawal of left hind limb in rats within 90 s were observed).

### Statistical methods

SPSS 19.0 (Chicago, IL, USA) was used for statistical analysis. The enumeration data were examined by X^2^. The measurement data were expressed as (X±s). *t* test was used for comparison of measurement data between two groups. F analysis was used for multigroup analysis. When *P*<0.05, the difference was statistically significant.

## Results

### General data

The body weight before and after modeling, length, age and glucose concentration in group A, B, C and control group were compared, and there was no significant difference between each group (Table 1).

**Table 1:** General data of mice in each group

***Group***	***Group A (n=20)***	***Group B (n=20)***	***Group C (n=20)***	***Control group (n=20)***	***F***	***P***
Body weight before modeling (g)	210.81±10.20	209.49±11.32	210.63±12.50	212.83±10.60	0.308	0.819
Body weight after modeling (g)	209.50±11.23	212.74±10.40	210.43±10.54	211.09±10.32	0.330	0.804
Length (cm)	19.03±1.72	19.95±1.61	19.89±1.43	19.74±0.09	1.894	0.138
Age (Week)	8.11±0.34	8.20±0.12	8.30±0.53	8.06±0.10	2.121	0.105
Glucose (mmol/L)	83.84±11.38	84.84±12.17	83.24±11.36	84.61±11.55	0.080	0.971

### Treatment effect

The average value in the control mice was taken as the standard (Spontaneous paw withdrawal 8.3 times; PWL 4.6 s; PWT 15.7 g), and the improvement of pain behavior in mice was used to evaluate the efficacy of drug therapy. Of the 20 mice in group A, the cancer pain of 11 mice was alleviated in varying degrees, the therapy was ineffective in 9 mice, and the effective rate was 55%. Of the 20 mice in group B, 12 were effective and 8 were ineffective, and the effective rate was 60.00%. Of the 20 mice in group C, 18 were effective and 2 were ineffective, and the effective rate was 90.00%. There were differences between the three groups, and the efficacy in group C was the best (*P*<0.05), and there was no difference in efficacy between group A and group B (Table 2).

**Table 2:** Effective rate of treatment of mice in each group

***Group***	***n***	***Effective rate of treatment***
Group A	20	55.00%
Group B	20	60.00%
Group C	20	90.00%^*^
X^2^		6.624
*P*		0.036

Note:

* represented that the effective rate of treatment in this group was significantly higher than that in the other two groups, and the differences were statistically significant (*P*<0.05)

### Comparison of physical improvement

Taking the average value in the control group as the standard (Area above threshold of struggle: 7348.6 mv·s; Accumulated immobile time: 103.6 s; Single accumulated immobile time: 60.5 s), the physical condition of mice in group A, B and C was evaluated. Of the 20 mice in group A, 5 were physically improved and the improvement rate was 25.00%. Of the 20 mice in group B, 6 were physically improved and the improvement rate was 30.00%. Of the 20 mice in group C, 13 were physically improved and the improvement rate was 65.00%. There was difference in the physical improvement among the three groups, and the improvement in the group C was the greatest (*P*<0.05) (Table 3).

**Table 3:** Comparison of physical improvement of mice in each group

***Group***	***n***	***Physical improvement rate***
Group A	20	25.00%
Group B	20	30.00%
Group C	20	65.00%^*^
X^2^		7.917
*P*		0.019

Note:

* represented that the physical improvement rate in this group was significantly higher than that in the other two groups, and the differences were statistically significant (*P*<0.05)

### Comparison of area of bone metastases of lung cancer in four groups

**Fig. 1:**
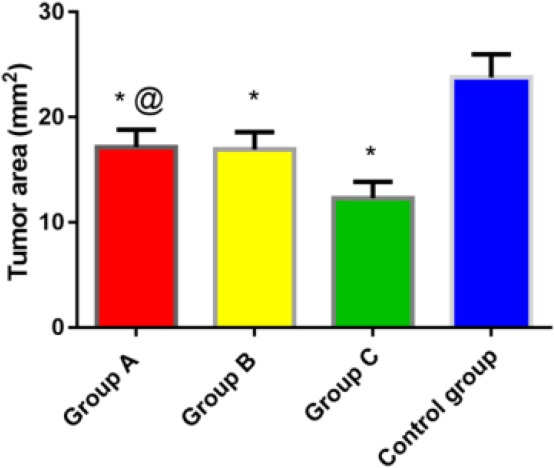
Comparison of area of bone metastases of lung cancer in four groups Note: *P<0.05 compared with the control group; ^#^*P*<0.05 compared with group A and group B

The observation of tumor growth in real time by Fluor Vivo imaging system during the experiment found that there were no metastases in other parts and organs. The tumor area in group A, B and C after 21 days of treatment was (17.16±7.37) mm^2^, (16.94±7.37) mm^2^, and (12.32±6.94) mm^2^, respectively, which was significantly lower than that in the control group (23.78 ±9.86) mm^2^, and the difference was statistically significant (*P*<0.05). The tumor area in group C was significantly lower than that in group A and group B, and the difference was statistically significant (*P*<0.05); There was no significant difference between group A and group B (Fig. 1).

### Comparison of tumor weight in four groups of mice after 21 days of treatment

**Fig. 2:**
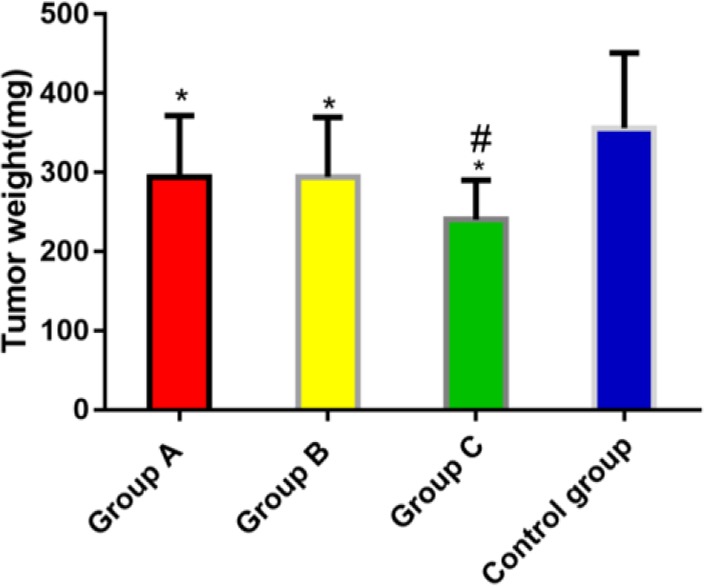
Comparison of tumor weight in each group of mice after treatment *P<0.05 compared with the control group; ^#^*P* <0.05 compared with group A and group B

The tumor weight in group A, B and C was (293.75 ±78.22) mg, (294.49 ±75.13) mg and (240.66 ±49.71) mg, respectively, which was significantly lower than that in the control group (356.09 ±95.12) mg, and the difference was statistically significant (*P*<0.05). The tumor weight in group C was significantly lower than that in group A and group B, and the difference was statistically significant (*P*<0.05). There was no significant difference between group A and group B (Fig. 2).

## Discussions

Lung cancer, the third largest malignant tumor in the world and the leading cause of cancer death in patients, seriously affects human health (17). The incidence of lung cancer is related to many factors. Age and smoking are the important causes of lung cancer deterioration or metastasis, and smoking is a main risk factor (18). Bone is the most common site for metastasis of lung cancer. The incidence of bone metastasis was about 40% in non-small cell lung cancer (NSCLC) patients, and more than 55% of the patients had bone tissue lesions at first visit (19). In this study, mouse model of bone metastasis of lung cancer was established to conduct an experimental study of paclitaxel combined with kanglaite in the treatment of bone metastasis of lung cancer in mice, and to analyze its value in the treatment of bone metastasis of lung cancer.

There are few reports on the efficacy of paclitaxel combined with kanglaite in bone metastasis of lung cancer. But the results of clinical studies on paclitaxel combined with kanglaite in breast cancer and other malignant diseases are similar to those of this study, both indicating that the effect of paclitaxel combined with kanglaite on the improvement of tumor was significantly higher than that of paclitaxel or kanglaite alone, which is a supporting evidence of the results of this study (20). The tumor area and tumor weight in group C were significantly lower than those in group A and group B, the difference was statistically significant. There’s no study on the specific tumor area and weight of bone metastases of lung cancer treated by paclitaxel combined with Kanglaite. However, there are still some related reports that kanglaite combined with other chemotherapeutic drugs was much more effective in the short-term treatment of lung cancer than kanglaite injection alone by comparing the objective response rate (ORR), Karnofsky Performance Status (KPS) and pooled risk ratio (RR) of nausea and vomiting (21, 22).

In this experiment, the number of modeling mice is not enough to be a large data, which may lead to occasional results, so this study is only a reference for later researchers. However, we will continue to carry out research on bone metastasis of lung cancer at a later stage, and follow the relevant research reports in real time to improve this research continuously.

## Conclusion

Paclitaxel combined with kanglaite is more effective than paclitaxel or kanglaite alone in improving bone metastasis of lung cancer and has an important significance in clinical treatment of bone metastasis of lung cancer.

## Ethical considerations

Ethical issues (Including plagiarism, informed consent, misconduct, data fabrication and/or falsification, double publication and/or submission, redundancy, etc.) have been completely observed by the authors.
